# Effects of early physical therapy on motor development in children with Down syndrome

**DOI:** 10.14744/nci.2020.90001

**Published:** 2022-04-18

**Authors:** Feyzullah Necati Arslan, Derya Gumus Dogan, Sinem Kortay Canaloglu, Senay Guven Baysal, Raikan Buyukavci, Mehmet Akif Buyukavci

**Affiliations:** 1 Division of Developmental and Behavioral Pediatrics, Department of Pediatrics, Necip Fazil City Hospital, Kahramanmaras, Turkey; 2 Division of Developmental and Behavioral Pediatrics, Department of Pediatrics, Inonu University Faculty of Medicine, Malatya, Turkey; 3 Department of Physical Medicine and Rehabilitation, Inonu University Faculty of Medicine,, Malatya, Turkey; 4 Department of Developmental and Behavioral Pediatrics, Malatya Training and Research Hospital, Malatya, Turkey

**Keywords:** Bayley III, Down syndrome, early physical therapy

## Abstract

**Objective:**

The objective of the study was to compare the motor development of children with Down syndrome (DS) who received physical therapy (PT) and did not receive PT, and to show the effect of PT programs started before the age of one on movement development.

**Methods:**

The study included aged between 6 and 42 months, 58 children with DS. Children with DS were divided into two groups as receiving PT and non-receiving PT. Children with DS who received PT were further divided into two groups according to the age of starting PT as before and after 1 year of age. Gross motor and fine motor development of the cases were evaluated with Bayley Scales of Infant and Toddler Development III.

**Results:**

Gross motor scaled scores (GM-SS: 3.88±3.46–1.67±1.23), fine motor scaled scores (FM-SS: 4.29±3.24–1.79±0.93), and composite scores (64.4±19.5–50.38±5.38) of PT group were statistically higher than the non-PT group (p<0.05). In addition, GM-SS (5.22±4.23–2.38±1.20), FM-SS; (5.61±3.85–2.81±1.37), and composite scores (72.33±23.85–55.56±5.7) of the cases who started PT before the age of one were statistically higher than those who started after the age of one (p<0.05).

**Conclusion:**

Our results revealed that PT especially when started early childhood under had a positive effect on the development of gross and fine motor in children with DS and provided a scientific basis for referring children with DS to PT programs before the age of one. Clinicians should recommend PT for children with DS in the early period.

**D**own syndrome (DS) is the most common chromosomal anomaly with a rate of 1 in 1000 and 1100 births worldwide [1]. Children with DS display similar physical features and often delays in cognitive, language, and motor developments, due to extra 21^st^ chromosome. Various abnormalities involving respiratory, cardiovascular, endocrine, gastrointestinal, hematological, immunological, musculoskeletal, genitourinary, and neurological systems may also accompany [2–4]. Life expectancy of DS individuals has increased significantly during the past 50 years [5]. Increases in life expectancy and quality of life have increased the importance of interventions aiming to enable children with DS.

Early childhood is a critical stage in a child’s development. Many skills in cognitive, motor, language, social, and emotional areas that enable children to interact with their environment develop in this period. Delayed motor development is of particular importance because it may cause delay in acquiring some skills in cognitive, emotional, and social areas [5–8]. In children with DS, hypotonia, ligament laxity, joint instability, muscle weakness, and issues of balance and coordination mechanisms cause delays in motor development [6, 9, 10]. Mean age and age range of walking among typically developing children are, respectively, 13 months and 9–17 months, whereas 24 months and 14–42 months in children with DS [3].

Balance can affect the development of motor skills, especially in childhood. Hypotonia and postural abnormalities lead to delays in the development of balance in children with DS. Since balance and motor functions are interrelated, they should be considered in physical therapy (PT) programs for children with DS [7]. It has been shown that, physical exercise and activity increase muscle strength and motor skills, in children with special needs in motor development as well as healthy developing children. PT programs should target developing basic motor skills such as walking, balance, and jumping as well as preventing future complications [5, 9, 10].

Most studies on the subject of motor development in children with DS are related to the age of acquisition of basic gross and fine motor skills [3, 6, 7, 11, 12]. In the meta-analysis by Ruiz-Gonzalez et al. [5], which included a large group of children with DS, it was pointed out that different PT intervention patterns were effective in improving the different motor functions, and PT was recommended for children with DS, to increase muscle strength and balance. However, in the literature, there is no study comparing the effects of initiation of PT in the early period (especially in the 1^st^ year) and later periods on motor development in these children.

Although the importance of early intervention is known, several challenges including medical problems in children with DS, families’ being poorly informed about DS, physicians’ delay in referring to PT in some cases, families’ being resident in rural areas, and problems peculiar to the health-care system cause delays in the initiation of PT.

The aim of this study is to compare gross and fine motor development in children with DS who received and who did not receive PT due to the reasons we mentioned, at an early age and to demonstrate the effect of starting PT programs in the early period (before 1 year of age) on motor development, to underline this problem and to raise awareness. In addition we investigated the effects of PT programs which started earlier (before 1 year of age) on motor development.

## Materials and Methods

The study included children between 6 and 42 months of age, who admitted to our Developmental Pediatrics Outpatient Clinic and were diagnosed with DS genetically, between April and June 2019. Fifty eight children with DS were included in the study. Children younger than 6 months and older than 42 months and those with premature birth, epilepsy, intracranial hemorrhage, hearing and vision loss were excluded from the study. Ethics Committee approval was obtained from Inonu University, Faculty of Medicine Local Ethics Committee (2019/7-16). Written consent was obtained from the families. This study was conducted following the principles of the Helsinki Declarations revised in 2013.

The socio-demographic data and concomitant medical conditions of children with DS were recorded. In addition, whether children with DS received PT before admission and if they had received PT, when it was started (before or after 1 year of age), how long and how frequently they had received were questioned.

Children with DS who were found not to receive any PT program were referred to the PTOutpatient Clinic. The PT program was started in patients with retardation of motor development. However, due to delays in admission, all children could not access PT programs at an early age.

Children with DS were divided into two groups as receiving PT and non-receiving PT. The cases who had been treated regularly at any rehabilitation center for at least 12 weeks were defined as received PT. Whether, these patients received that PT is detected depending on the statements of the parents.

Children with DS who received PT were further divided into two groups according to the age of starting PT as before and after 1 year of age. In our country, currently, routine PT programs are administered on average 2 days per week and 45 min for each session. The PT program is individualized according to age and motor development stage of the patients. The program aims at maintaining the balance and developing a variety of neuromuscular components including muscle strength, physical coordination, and functional movements in the lower and upper extremities, for all patients. In the study, the group receiving PT did not receive any additional therapy, other than PT applied in rehabilitation centers.

All patients included in the study were evaluated in terms of gross and fine motor development using the Bayley III scale (Infancy and Early Childhood Developmental Assessment Scale).

### Evaluation Tool

In this study, Bayley III scale was preferred as an evaluation tool. Bayley III is a gold standard assessment scale, that measures fivedifferent areas including cognitive, expressive language, recipient language, fine movement, and rough movement in children aged 1–42 months, with proven reliability and validity in determining the delays in assessment areas and determining the need for intervention. This scale was standardized with a sample of 1700 children in the United States, with a high reliability (0.80–0.87) and validity (0.86–0.93) [13, 14].

The set of tasks applied may vary depending on the children’s age and performance. Each task is scored as 1 (full completion) or 0 (partial completion or not able to do at all). For this study, performance was measured by means of scaled scores, which are standardized scores based on the instrument’s normative sample. The scaled score varies from 1 to 19 (mean=10; standard deviation [SD]=3) and performance within the average should be between 7 and 13 [13].

Bayley III motor development test results were expressed as mean score of 100 points and SDof 15 points. Composite scores for motor development between 70 and 84 (−1 to −2 SD) are considered to be abnormal, and composite scores below 69 (<2 SD) indicate severe developmental delay [13].

Bayley III evaluation was carried out by appropriately trained and experienced researchers. Gross motor scaled score (GM-SS), fine motor scale score (FM-SS), and composite motor scores were found in patients using Bayley III.

### Statistical Evaluation

Statistical evaluation of the data was performed using Statistical Package for the Social Sciences (SPSS 17) software (SPSS Inc, Chicago, IL, USA). In the power analysis of the study, when alpha: 0.05 and 1-beta (power): 0.80, if the mean difference in the detected composite scores was 9 units in the evaluation of motor development of the receiving PT program and not receiving PT program subgroups the of children with DS with Bayley III, it was calculated that at least 21 cases should include in the study. Shapiro–Wilk test was used to determine normal distribution. Mann–Whitney U test wasused to test the differences. Mean and SD values were given as descriptive statistics. Categorical variables were examined using Pearson’s Chi-squared test. The results were considered statistical significance if p values were <0.05.

## Results

Thirty (51.7%) male and 28 (48.3%) female patients were included in the study. The average age in the PT receiving group (34 cases) was 21.1±8.2 months and 17.2±8.8 months in non-PT receiving group. There was no significant difference in terms of mean age, and mother and father age in both groups (p>0.05). The details are given in Table 1.

**Table 1.** Socio-demographic data

Variables	PT receiving group (n=34) Mean±SD	Non PT receiving group (n=24) Mean±SD	p
Chronological age (months)	21.1±8.2	17.2±8.8	(p=0.09)
Sex (male/female)	21/13	9/15	(p=0.12)
Maternal age (years)	35.2±6.5	34.4±4.5	(p=0.58)
Paternal age (years)	38.8±6.4	39.08±6.7	(p=0.88)

PT: Physical therapy; SD: Standard deviation.

The socio-demographic data of the cases aregiven in Table 1. The incidence of congenital heart disease (CHD) was 53.4% (n=31). In terms of CHD association, there was no significant difference between PT receiving group (n=17) and non-PT receiving group (n=14) (p=0.12).

**Figure 1.**
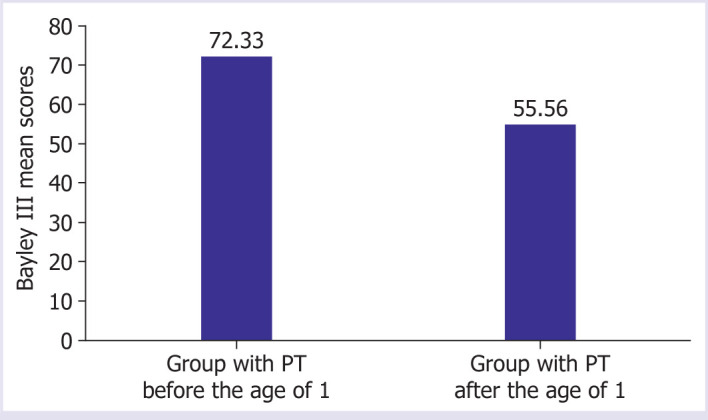
Composite motor scores between group with PT before the age of 1 and group with PT after the age of 1.

There was a significant difference between the PT receiving group and the non-PT receiving group in terms of GM-SS, FM-SS and motor composite scores (p<0.05). Details are given in Table2. The mean duration of the PT period was 42.1±28.4 weeks, at least 12 weeks and maximum 120 weeks. We found significant differences between the subgroups who started PT before the age of one and after the age of one in terms of GM-SS, FM-SS, and motor composite scores (p<0.05). The details are given in Table 3 and Figure 1. The mean PT duration of the subgroup who started PT before 1 year of age was 41.2±29.6 weeks, and the mean PT period of the group who started PT after 1 year of age was 43.2±28.3 weeks. It was observed that children who started PT before 1 year of age in all groups had higher scores in GM-SS, FM-SS, and motor composite scores than the other groups.

**Highlight key points**

• Children with Down syndrome (DS) experience varying degrees of motor and global developmental delays that reduce their ability to participate in life.
• Physical therapy (PT) has a positive effect on the development of gross and fine motor skills in children with DS.
• Our study showed that, starting PT especially before 1 years of age in children with DS has an extra positive effect on gross, fine, and total motor development.

**Table 2.** Gross motor scaled score (GM-SS), fine motor scale score (FM-SS), and composite motor scores between PT receiving group and non-PT receiving grou

Variables	PT receiving group (n=34) Mean±SD	Non PT receiving group (n=24) Mean±SD	p
GM-SS	3.88±3.46	1.67±1.23	<0.001
FM-SS	4.29±3.24	1.79±0.93	<0.001
Composite motor scores	64.4±19.5	50.38±5.38	<0.001

PT: Physical therapy; SD: Standard deviations; GM-SS: Gross motor scaled score; FM-SS: Fine motor scale score. Statistical significance p values <0.05.

**Table 3.** Gross motor scaled score (GM-SS), fine motor scale score (FM-SS), and composite motor scores between group with PT before the age of 1 and group with PT after the age of 1

Variables	Group with before the the age of 1 (n=18)	Group with PT after the age of 1 (n=16)	p
GM-SS (Mean±SD)	5.22±4.23	2.38±1.20	0.02
FM-SS (Mean±SD)	5.61±3.85	2.81±1.37	0.03
Composite motor scores (Mean±SD)	72.33±23.85	55.56±5.71	0.01

PT: Physical therapy; SD: Standard deviations; GM-SS: Gross motor scaled score; FM-SS: Fine motor scale score. statistical significance p values <0.05.

## Discussion

The results of this study revealed that PT programs which started under 42 months and especially, before the age of 1 year in children with DS positively affected gross, fine, and total motor development.

Children discover the outside world byholding, grasping, tasting, and examining objects around them, in the early period. Ability to walk in balance allows a child to use his hands freely and to explore the world independently [7]. DS is one of the most common causes of developmental delay [15]. Children with DS experience varying degrees of motor and global developmental delays that reduce their ability to participate in life. The acquisition of motor skills during infancy facilitates cognitive, social, physical, and emotional development and child-environment interactions [16]. Motor experiences affect babies’ interest in both the physical and social world. In addition, it has been shown that, following the beginning of vertical movement, infants more interact with objects and their mothers [17].

Interventions to reduce delays in motor development may positively affect the development of DS babies [16]. There are many different PT interventions targeting children with DS. In a meta-analysis by Ruiz-Gonzalez et al. [5], it was reported that different PT interventions were effective in improving the outcome of different motor functions in DS patients and that it should be recommended to increase muscle strength and balance in these children. Similar to the literatureon the efficacy of PT in children with DS, in our study, the motor development of the DS patients who received PT was significantly better than those who did not receive PT.

Interventions based on resistance exercises are effective in the development of upper and lower extremity muscle strengths. It has been proven that the balance increases with the maximum efficiency of the upper and lower extremities, especially the mediolateral displacement of the center of gravity [5]. Malak et al. [18], in their study on 79 children with DS, found that equilibrium functions and motor skills were closely interrelated, especially in the first 3 years of life. Early PT interventions in DS babies are expected to enable the babies to perform functional activities, explore the environment, move towards commands, and to minimize the changes in DS-specific musculoskeletal system [12]. The delay in motor development occurs at about 4 months of age. This delay is especially evident in skills that require high levels of muscle activity against gravity. It seems that DS babies need more time to develop an acquired skill [16, 19].

In studies suggesting that the PT in DS baby should be started up to the 3^rd^ month to have adequate stimuli at different postures; it was reported that if the stimulation is initiated early, the time required to develop the movement skills needed by DS babies becomes shorter and especially motor acquirements against gravity are facilitated [16, 19].

In our study, each child was included in appropriate PT programs, which provides certain standards according to their ages to examine the effect of PT on the motor development.

It was also aimed to raise awareness of the caregivers of the children by revealing the difference between the children with DS who received early PT support and who did not, in terms of motor developmentinclusion of children with DS in Bayley III reliability test also supported the use of Bayley III in measuring skills in children with DS. The Bayley III standardization study included 90 children with DS, aged between 5 and 42 months with similar demographic characteristics. The mean motor composite score of the children with DS was 62.3 and it was found to be well below the mean motor composite score (102.3) of the control group [13]. In our study, the motor composite score of the subgroup who started PT before 1 year of age was 10 points higher than the DS group in the standardization study, and the group that started PT after the age of one was 7 points behind the DS group in the standardization study. We also found that, GM-SS, FM-SS, and motor composite scores of the subgroup who started PT before the age of one were significantly higher than the subgroup who started PT after the age of one. These results suggest that starting the PT program before the age of one is more effective.

CHD, which is one of the problems that may affect motor development, is seen in 41–56% of children with DS [20]. In our study the frequency of CHD was similar to the literature and there was no significant difference between the subgroups receiving PT and non-receiving PT in terms of accompanying CHD.

Although our study included more cases than in previous studies, a limitation of the study was that it was conducted in a single center and the sample size was still relatively small. There is need for studies investigating the effectiveness of PT applied before the age of one in children with DS, conducted in more number of centers which apply standard therapies, on larger number of cases, with longer follow-up.

Our results show that PT has a positive effect on the development of gross and fine motor skills in children with DS. It is striking that starting PT before 1 years of age has an extra positive effect on gross and fine motor development. Health systems that will enable children with DS to reach PT in the early period should be implemented. In addition, the sensitivity of clinicians should be increased not to delay interventions. The results of our study have provided a scientific basis for the health workers working with children with DS to direct the children and families to PT programs at the earliest age, especially before the age of 1 year.
